# Crossing and Anticrossing of Exchange‐Coupled Molecular Spin Excitation Energy Levels

**DOI:** 10.1002/smll.202412703

**Published:** 2025-05-27

**Authors:** Lorenz Meyer, Maximilian Kögler, Robert Henninger, Nicolas Néel, Jörg Kröger

**Affiliations:** ^1^ Institut für Physik Technische Universität Ilmenau D‐98693 Ilmenau Germany

**Keywords:** exchange interaction, inelastic electron tunneling spectroscopy, metallocene, scanning tunneling microscopy, spin excitation

## Abstract

The superconducting Pb tip of a scanning tunneling microscope (STM) is functionalized with a single nickelocene (Nc) molecule and approached to individual Nc molecules embedded in a molecular island adsorbed on the (111) surface of the conventional superconductor Pb. Excitations of the tip and surface molecular spins that are coupled across the vacuum barrier via the magnetic exchange interaction are explored by inelastic electron tunneling spectroscopy (IETS) from the far tunneling to the contact range of intermolecular distances. Depending on the tilt angle of Nc at the microscope probe, single spin and double spin flip energy levels cross for the straight Nc‐terminated tip and exhibit avoided crossing for the tilted configuration. The crossing‐to‐anticrossing transition of spin excitation energy levels can be reproduced by simulations based on a spin Hamiltonian where the magnetic anisotropy tensor of the Nc tip is rigidly rotated.

## Graphical abstract

Orientation matters: The excitation energy levels of exchange‐coupled spins of two nickelocene molecules anchored to superconducting electrodes are explored via inelastic electron tunneling spectroscopy. Depending on the tilt angle of the single‐molecule magnetic probe the levels cross or exhibit avoided crossing.

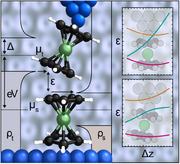

## 1 Introduction

Single magnetic atoms and molecules have been attracting substantial interest lately because they represent at the ultimate scale promising building blocks for memory storage,^[^ ^1^, ^2^ ^]^ quantum computing,^[^ ^3^, ^4^, ^5^ ^]^ molecular spintronics,^[^ ^6^, ^7^, ^8^, ^9^, ^10^ ^]^ and spin sensors.^[^ ^11^, ^12^, ^13^, ^14^, ^15^ ^]^ From a fundamental perspective the spectroscopic observation of quantum spin excitations is key to the understanding of the mechanisms and principles underlying magnetic interactions.

The STM appears to be a particularly suitable tool for exploring the physics of coupled quantum spins. Besides its capabilities of imaging with atomic resolution and the atom‐by‐atom manipulation of matter, it offers the spectroscopy of excitations with high energy resolution. Previously, the coupling of orbital and spin magnetic moments were explored through their impact on electronic orbitals,^[^ ^16^, ^17^ ^]^ the Kondo effect,^[^ ^18^, ^19^, ^20^, ^21^, ^22^, ^23^, ^24^ ^]^ magnetoresistive effects,^[^ ^25^, ^26^, ^27^, ^28^, ^29^, ^30^, ^31^, ^32^ ^]^ spin excitations,^[^ ^12^, ^33^, ^34^, ^35^, ^36^, ^37^, ^38^ ^]^ Yu‐Shiba‐Rusinov states,^[^ ^39^, ^40^, ^41^, ^42^ ^]^ and magnetization properties.^[^ ^43^, ^44^ ^]^

Compared to experiments where atomic or molecular spins reside at surfaces and, hence, the mutual distances and orientations of spins are determined by the crystallographic environment, the intentional decoration of the STM tip with a single‐atom or single‐molecule spin offers appealing advantages. The separation between a spin at the tip and on the surface can be tuned in an almost continuous manner over a wide range covering well decoupled spins in the tunneling and touching spins in the contact region. Moreover, the spin orientation of the tip apex can be controlled to some extent by modifying the atomic structure of the tip apex^[^ ^45^ ^]^ and determining the tilt angle of molecular probes.^[^ ^46^, ^47^, ^48^, ^49^ ^]^ Such experiments were performed to explore, e. g., the magnetic coupling between two atoms.^[^ ^50^, ^51^ ^]^ More recently, metallocene molecules terminating the STM tip were demonstrated to act as sensitive spin sensors.^[^ ^47^, ^48^ ^]^ The intriguing property of metallocene molecules is the preservation of the gas‐phase spin ground state upon adsorption and its engineering by the central metal atom.^[^ ^52^, ^53^ ^]^ Pioneering work showed the impact of the interaction with magnetic adsorbates on the metallocene spin excitation spectrum.^[^ ^47^, ^48^, ^54^, ^55^ ^]^ In the particular case of Nc (Ni (C_5_H_5_)_2_) decorating an STM tip, the lifting of the spin energy level degeneracy was shown to crucially depend on the neighboring exchange field.^[^ ^47^, ^55^ ^]^

**Figure 1:**
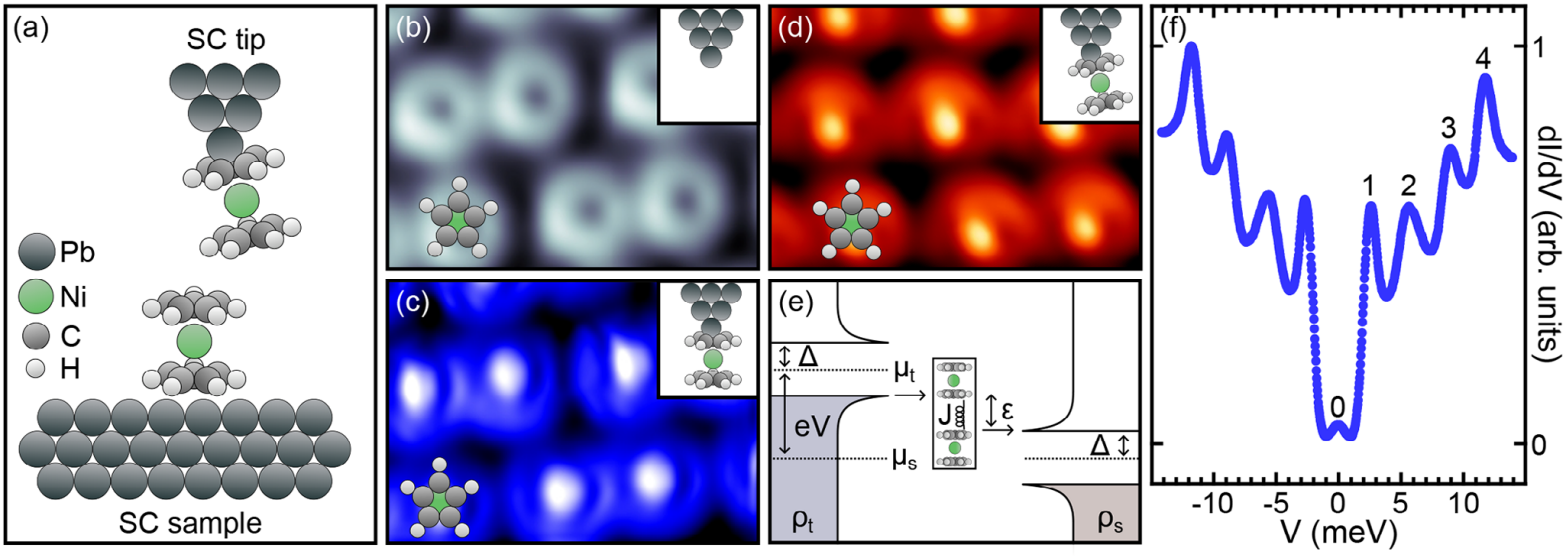
a) Sketch of the STM junction comprising a superconductor (SC) Pb tip terminated by a single Nc, and a single Nc adsorbed on the SC Pb(111) sample. b) STM image of a few Nc molecules embedded in a molecular island recorded with a pristine Pb tip (−100 mV, 30 pA, 2.8 nm × 1.8 nm). c,d) As (b) acquired with a (c) straight and (d) tilted Nc tip (100 mV, 30 pA (c), −100 mV, 25 pA (d)). e) Illustration of inelastic electron tunneling from an SC tip with BCS DOS ϱ_t_ to an SC sample with BCS DOS ϱ_s_ at *T* = 0. A spin excitation with energy ε occurs for sample voltages |*V*| ⩾ (2Δ + ε)/e. f) Representative d*I*/d*V* spectrum acquired with a tilted Nc tip above adsorbed Nc (labels 0–4 are explained in the text). After deactivating the feedback loop at 100 mV and 60 pA the tip was approached toward the surface by Δ*z* = 340 pm.

In the work presented here, the interaction between two Nc molecular spins, one terminating a superconducting Pb tip and the other adsorbed on Pb(111) (**Figure** **1**a), is probed depending on their mutual distance. Each Nc exhibits a spin triplet (spin quantum number *S* = 1) ground state with the easy plane parallel to the cyclopentadenyl (C_5_H_5_, Cp) groups.^[^ ^47^, ^52^, ^54^, ^56^, ^57^ ^]^ The experimental studies were stimulated by the open question as to the influence of the Nc tilt angle at the tip on the evolution of excitations in the Nc–Nc coupled spin system. While indications were reported for a normal‐metal Nc–Nc junction on Ag(110)^[^ ^47^ ^]^ and on Ag(100),^[^ ^57^ ^]^ a systematic analysis of the impact of the mutual Nc spin orientation on excitation spectra has been missing to date. Probing the spin excitation of the exchange‐coupled Nc molecules via IETS from the tunneling to the contact range, the evolution of spin excitation energy levels is traced with decreasing Nc–Nc distance and, thus, increasing magnetic exchange interaction. The improved energy resolution in spectroscopy owing to a superconducting STM tip^[^ ^39^, ^58^ ^]^ reveals a level crossing of single spin and double spin flips when the magnetic easy planes of Nc at the tip and Nc on Pb(111) are parallel, i. e., for straight Nc tips. In contrast, the level crossing is avoided when these planes are inclined against each other, i. e., for tilted Nc tips. These observations can be reproduced with a simple model spin Hamiltonian. In addition, the validity of approaches to transition matrix elements affecting the strengths of spin excitation processes is discussed.

## 2 Results and Discussion

### 2.1 General Approach to the Intermolecular Exchange Coupling

Deposition of Nc on cold (≈80 K) Pb(111) gives rise to an ordered molecular superstructure, where dimers of Nc are assembled in islands (Figure 1b), in agreement with a previous report.^[^ ^56^ ^]^ The upright adsorption of Nc on Pb(111) (Figure 1a) manifests itself in STM images acquired with a Pb tip by the ringlike protrusion with a central depression, which is assigned to the top Cp moiety.^[^ ^56^ ^]^ The STM data of adsorbed Nc change strongly when an Nc‐decorated tip is used. In particular, the central depression observed with a Pb tip turns into a marked protrusion (Figure 1c,d). Straight Nc tips, whose long molecular axis encloses the angle ϑ = 0° with the surface normal, give rise to the top Cp group of adsorbed Nc appearing with an almost uniform contrast (Figure 1c), while it is nonuniform for tilted (ϑ > 0°) Nc tips (Figure 1d). Such STM images can therefore be used to qualitatively discriminate straight from tilted Nc tips. By analyzing appropriate cross‐sectional profiles of Nc molecules imaged with an Nc‐decorated tip the tilt angle can be estimated (Supporting Information, Figure S1). Due to the entanglement of geometric and electronic properties in STM images such an estimate has tentative character, however. Notably, the conclusions of this work rely on the presence of straight and tilted tips, rather than on a specific value of the finite tilt angle.

The following IETS experiments aim at detecting spin excitations of the exchange‐coupled molecules. For the interpretation of the resulting spectra, the superconducting state of tip and sample must be considered, as schematically illustrated in Figure 1e. The Bardeen–Cooper–Schrieffer (BCS) density of states (DOS) of the tip (ϱ_t_) and the sample (ϱ_s_) is depicted for vanishing temperature (*T* = 0). Because of missing quasielectron states in the energy range *E*_F_ ± Δ (*E*_F_: Fermi energy, 2Δ: width of the BCS energy gap) the sample voltage (*V*) must be at least |*V*| = (2Δ + ε)/e (e: elementary charge) for a spin transition with energy ε to occur. A typical spectrum of the differential conductance (d*I*/d*V*, *I*: current) acquired with an Nc tip above the Cp moiety is presented in Figure 1f. The peaks 1–4 and their partners at symmetric negative *V* are due to the coherence peaks of the BCS energy gap (1) and spin excitations (2–4). Rather than steplike variations of d*I*/d*V*, which are often observed for inelastic excitations in the case of normal‐metal tips and substrates, the spin excitation signals take the form of peaks in the case of superconducting electrodes, which reflects their origin as replicas of the BCS coherence peaks (Figure 1e). The tiny peak labeled 0 is centered at zero sample voltage and assigned to thermally excited quasielectron tunneling at the finite temperature (5 K) of the experiments. While some variation of peak heights and positions was observed across the adsorbed molecule, similar to earlier findings for Nc on Ag(110),^[^ ^47^ ^]^ the evolution of spin excitation energy levels with the Nc–Nc distance, which is the focus of this work, remained invariant (Supporting Information, Figure S2). The spectroscopic experiments unveiled that the optimal resolution of spin excitations could be achieved by placing the Nc tip above an off‐center site of adsorbed Nc. Spectra as the one depicted in Figure 1f are discussed next for straight and tilted tips as well as for different intermolecular separations. Owing to their symmetry with respect to *V* = 0, the spectra are shown for positive *V* only.

### 2.2 Modeling of Spin Excitation Spectra

In order to simulate IETS data and, thereby, describe experimental spectra as the one in Figure 1f, the following methods were applied. The tunneling current is assumed to be a superposition of an elastic (*I*_e_) and inelastic (*I*_i_) contribution, i. e., *I* = *I*_e_ + *I*_i_. The elastic term reads

(1) $$I_{e}\left(V\right)\propto \int \left[f_{t}\left(E-eV\right)-f_{s}\left(E\right)\right]\varrho_{t}\left(E-eV\right)\varrho_{s}\left(E\right)\;dE$$

with *f*_t_ and *f*_s_ the tip and sample Fermi‐Dirac function, respectively, evaluated at energy *E* referred to the electron chemical potential μ. These functions contain the tip and sample temperature *T*_t_ and *T*_s_. The experimental spectra evidence the absence of Andreev^[^ ^59^, ^60^, ^61^ ^]^ and Yu–Shiba–Rusinov^[^ ^62^, ^63^, ^64^ ^]^ bound states and, therefore, tip and sample DOS ϱ_t_ and ϱ_s_ are captured by the BCS expression

(2) $$\varrho_{k}\left(E\right)=\text{sign}\left(E\right)\cdot ℜ\left[\frac{E-i\gamma_{k}}{\sqrt{{\left(E-i\gamma_{k}\right)}^{2}-\Delta_{k}^{2}}}\right]$$

with k = t, s and where sign(*E*) = ±1 for *E* above (+) and below (−) μ, ℜ denotes the real part and γ the Dynes parameter reflecting the finite quasiparticle lifetime.^[^ ^65^ ^]^ The transmission factor for the tunneling process is omitted in Equation (1) because the relevant sample voltages for exploring the spin excitations in constant‐height spectra are low compared to the electrode work functions, which justifies the assumption of a nearly constant transmission factor. To include inelastic processes at different energies ε, the change of the tunneling junction conductance with strength *h*_ε_ is considered in *I*_i_ as

(3) $$I_{i}\left(V\right)\propto \sum_{\varepsilon }h_{\varepsilon }\int \left[f_{t}\left(E-eV-\varepsilon \right)-f_{s}\left(E\right)\right]\varrho_{t}\left(E-eV-\varepsilon \right)\varrho_{s}\left(E\right)\;dE$$

This approach to *I*_i_ takes the missing quasielectron states inside the BCS energy gap into account and, thus, excludes initial and final states for the inelastic tunneling electron in this energy range. Moreover, it explains the coherence peak replica character of the line shape of inelastic transitions in d*I*/d*V* spectra. To obtain an expression for the experimentally measured d*I*/d*V* data, the simulated *I*(*V*) is numerically differentiated and convoluted with a function that appropriately takes the modulation broadening into account^[^ ^66^, ^67^ ^]^ (Supporting Information). In order to minimize the number of fit parameters, the values of Δ_t_, Δ_s_, γ_t_, and γ_s_ were extracted from a fit of the modulation‐broadened d*I*_e_/d*V* to experimental spectra of the BCS energy gap at the known experimental temperature *T*_t_ = 5 K = *T*_s_. The resulting Δ_t_ = 1.10 meV, Δ_s_ = 1.15 meV (in agreement with expectations^[^ ^68^ ^]^), γ_t_ = 20 µV and γ_s_ = 5 µV were then fixed for the further analysis of spin excitation spectra.

**Figure 2:**
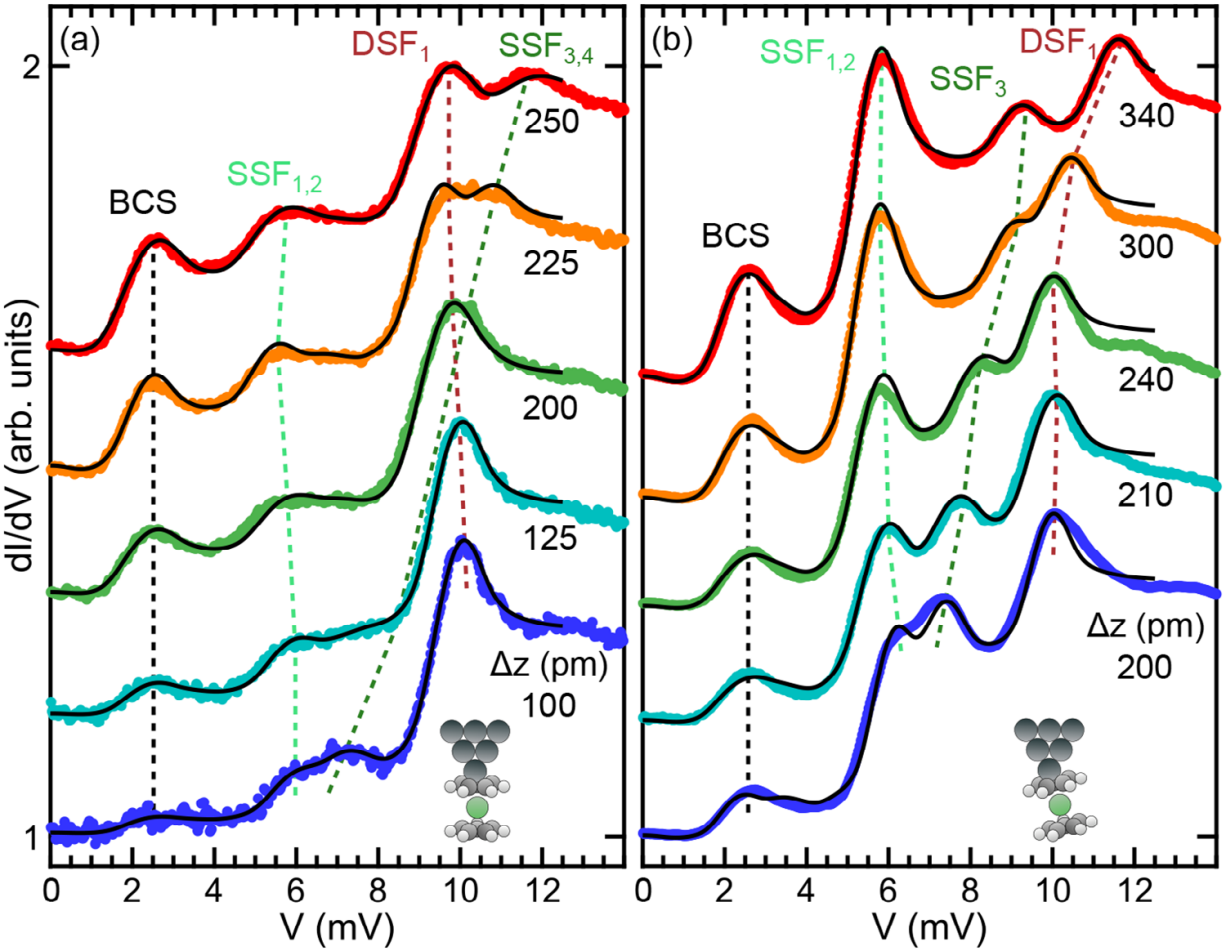
a) Collection of vertically offset d*I*/d*V* spectra (dots) acquired with a straight Nc tip laterally offset from an adsorbed Nc molecule with indicated tip excursion Δ*z*. The feedback loop parameters 100 mV and 60 pA define Δ*z* = 0. Solid lines depict fits to the data. Dashed lines guide the eye along the evolution of the BCS coherence peak and the inelastic transitions SSF_1 − 4_, DSF_1_ explained in the text. Inset: Sketch of the Nc tip geometry. b) Same as (a) for a tilted Nc tip. The spectra in (a) and (b) are normalized to their respective maximum.

**Figure** **2** shows experimental d*I*/d*V* data (dots) obtained with a straight (Figure 2a) and a tilted (Figure 2b) Nc tip for decreasing (from bottom to top) tip–surface distance, which is reflected by the increasing tip excursion Δ*z* marking each data set. The solid lines depict the simulated d(*I*_e_ + *I*_i_)/d*V* which best match the experimental data. From these fits, excitation energies ε and the associated inelastic signal strengths *h*_ε_ are extracted and used for the analysis discussed below, which will likewise clarify the assignment of spectral features to single spin flip (SSF) and double spin flip (DSF) excitations. Most importantly, while for the straight tip the SSF_3, 4_ and DSF_1_ excitation energy levels cross (see dashed lines indicating the evolution of spectral features with the tip excursion) this crossing is avoided for the tilted tip.

### 2.3 Evolution of Spin Excitation Energies with the Intermolecular Distance

**Figure 3:**
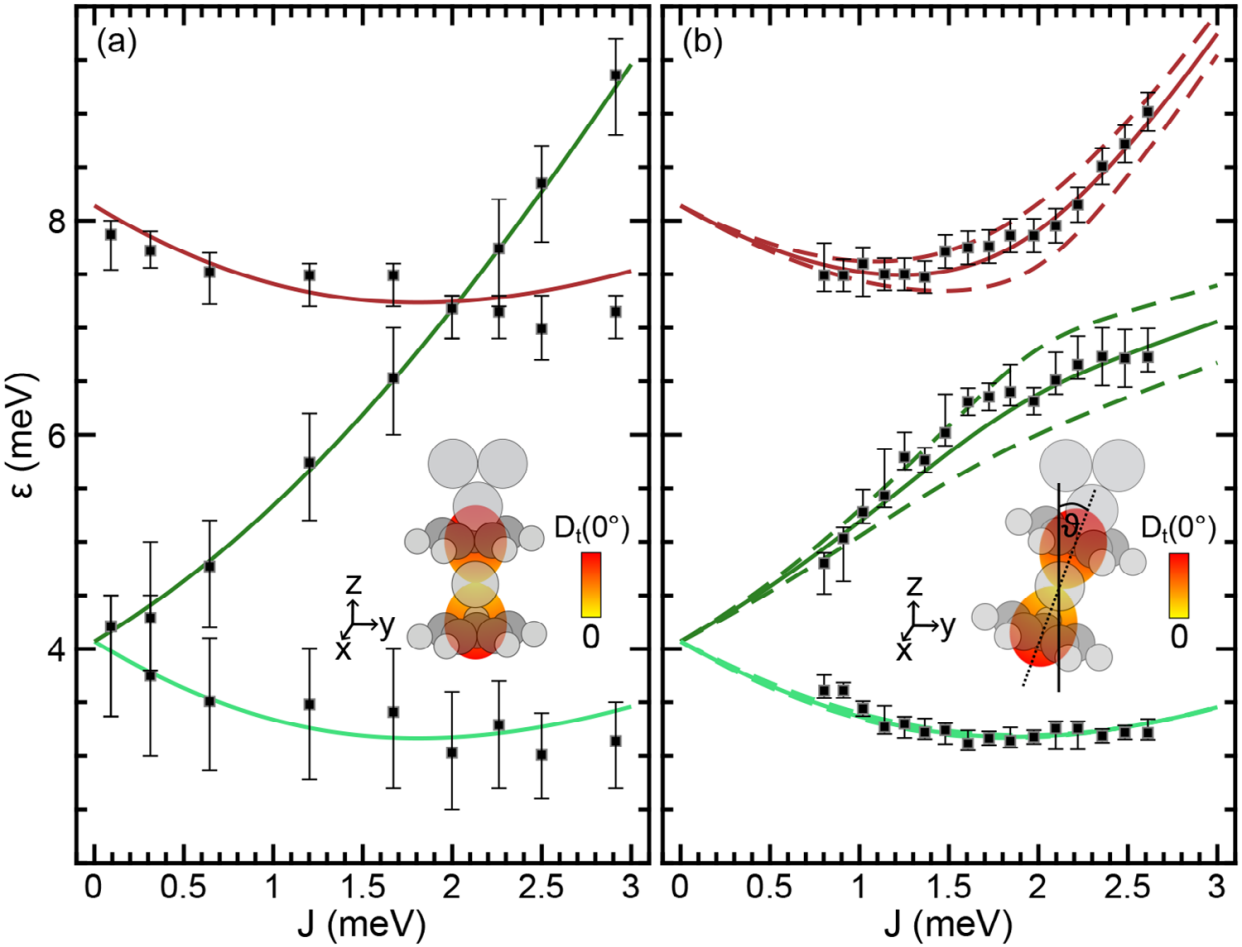
a) Spin excitation energies (squares) probed with a straight Nc tip and their evolution with *J* obtained from simulations (solid lines). The uncertainty margins reflect the 1 % decrease of the reliability factor underlying the least‐squares fits in Figure 2. Inset: Illustration of the quadric (dumbbell) associated with the tip magnetic anisotropy tensor together with a straight Nc tip. b) Same as (a) for a tilted Nc tip. Solid lines reflect simulation data obtained for ϑ = 21°, dashed lines illustrate the impact of tilt angle variations of Δϑ = ±3°.

**Figure** **3** presents the spin excitation energies obtained from fits to d*I*/d*V* data (Figure 2) as a function of the intermolecular magnetic exchange coupling *J* for a straight (Figure 3a) and a tilted (Figure 3b) Nc tip. The crossing (anticrossing) of energy levels for ϑ = 0° (ϑ ≠ 0°) becomes clearly evident. The aim of this section is the description of the data depicted in Figure 3 on the basis of an appropriate spin Hamiltonian, which includes the translation of the measured tip excursion Δ*z* to the magnetic exchange interaction *J* between the Nc molecules.

The modeling of the dependence of spin excitation energies on the magnetic exchange interaction follows from the eigenvalue analysis of the spin Hamiltonian^[^ ^11^, ^69^, ^70^ ^]^

(4) $$\hat{H}={\hat{H}}_{D}+{\hat{H}}_{J}$$

with

(5) $$\begin{array}{ccc} {\hat{H}}_{D}= & & {\hat{S}}_{t}^{T}\cdot D_{t}\left(\vartheta \right)\cdot {\hat{S}}_{t}+{\hat{S}}_{s}^{T}\cdot D_{s}\cdot {\hat{S}}_{s}=\sum_{\alpha ,\beta }{\hat{S}}_{t\alpha }D_{t\alpha \beta }\left(\vartheta \right){\hat{S}}_{t\beta }\\ & & +\sum_{\alpha ,\beta }{\hat{S}}_{s\alpha }D_{s\alpha \beta }{\hat{S}}_{s\beta } \end{array}$$

(α, β ∈ {*x*, *y*, *z*}) describing the magnetic anisotropy energy of Nc at the tip and on the surface, which is characterized by the anisotropy tensors **D**_t_(ϑ) and **D**_s_. For the straight (ϑ = 0°) Nc tip as well as for adsorbed Nc, all entries of **D**_t_(0°) and **D**_s_ are zero except for *D*_t*zz*_(0°) ≡ *D*_t_(0°) and *D*_s*zz*_ ≡ *D*_s_. The quadric of **D**_t_(0°) is illustrated in the inset to Figure 3a. The tilted (ϑ ≠ 0°) Nc tip is modeled here by the anisotropy tensor **D**_t_(0°) rigidly rotated about the *x*‐axis which, due to the system symmetry, is chosen arbitrarily within the easy plane, i. e.,

(6) $$D_{t}\left(\vartheta \right)=R^{T}\left(\vartheta \right)\cdot D_{t}\left(0^{\circ }\right)\cdot R\left(\vartheta \right)$$

with **R**(ϑ) the associated rotation matrix. The quadric of **D**_t_(ϑ) is depicted in the inset to Figure 3b. The components of the spin operators ${\hat{S}}_{t}$ and ${\hat{S}}_{s}$ read

(7) $$\begin{array}{c} {\hat{S}}_{t\alpha }={\hat{s}}_{t\alpha }⊗1_{s} \end{array}$$

(8) $$\begin{array}{c} {\hat{S}}_{s\alpha }=1_{t}⊗{\hat{s}}_{s\alpha } \end{array}$$

with ${\hat{s}}_{t}$, ${\hat{s}}_{s}$ the spin operators for *S* = 1 and $1_{t}$, $1_{s}$ the 2*S* + 1‐dimensional unity matrices. The operation ⊗ denotes the Kronecker product and gives rise in the current situation to a 9 × 9 matrix representation of $\hat{H}$. The second term of $\hat{H}$ (Equation (4)) models the magnetic exchange interaction between the Nc molecules and is expressed as

(9) $${\hat{H}}_{J}={\hat{S}}_{t}^{T}\cdot J\cdot {\hat{S}}_{s}=J{\hat{S}}_{t}\cdot {\hat{S}}_{s}$$

The right side of Equation (9) results from the assumed simplified magnetic exchange tensor of the form J=J1 with 1 the 3 × 3 unity matrix, which reflects a spatially isotropic Heisenberg interaction. This assumption is in good agreement with previous reports.^[^ ^47^, ^57^ ^]^

The model Hamiltonian contains the fit parameters *D*_t_(0°), *D*_s_, ϑ, and *J*. The following discussion shows that the first three of these parameters can be fixed on the basis of experimental observations, leaving *J* as the single fit parameter of the model. To this end, d*I*/d*V* spectra acquired with a straight Nc tip, that is, ϑ = 0°, in the far tunneling range of Nc–Nc separations, i. e., *J* ≈ 0, give rise to two spin excitations. The low‐energy (high‐energy) excitation is assigned to a SSF (DSF) process. Eigenvectors of $\hat{H}$ (Supporting Information, Figure S3) show that a SSF describes the flip of one of the Nc spins out of the easy plane to the hard axis, i. e., *M*_*S*_ = 0 transitions to *M*_*S*_ = 1 or to *M*_*S*_ = −1, while a DSF denotes the flip of both Nc spins. When both Nc spins are excited they point in opposite directions along the hard axis because of angular‐momentum conservation in the inelastic tunneling event.^[^ ^47^, ^52^ ^]^ The fit of such spectra (with ϑ = 0° and *J* = 0) results in *D*_t_(0°) = *D*_s_ = 4.0 ± 0.2 meV, which agrees with previous observations.^[^ ^47^, ^48^, ^49^, ^52^, ^56^, ^57^ ^]^

Besides *D*_t_(0°) and *D*_s_, the Nc tilt angle can be inferred from the observed anticrossing behavior of spin excitation energy levels. Figure 3b shows that SSF and DSF energy levels which cross for ϑ = 0° (Figure 3a) avoid crossing for ϑ ≠ 0°. The minimum energy difference Δε between these levels can experimentally be determined as Δε = 1.4 ± 0.1 meV. This energy difference is approximately related to the Nc tilt angle via Δε = [*D*_t_(0°)sin 2ϑ]/2 (Supporting Information). By comparing the measured Δε with the numerical solution (Supporting Information, Figure S4), the tilt angle results in 21 ± 3°. This value is considered more reliable than the tentative estimation based on cross‐sectional profiles of STM data.

The independently obtained values of *D*_t_(0°), *D*_s_, and ϑ are retained for describing the overall evolution of spin excitation energies ε as a function of the tip excursion Δ*z*, which is now solely determined by *J*. In particular, the tilt angles, i. e., ϑ = 0° for the straight and ϑ = 21° for the tilted Nc tip, are assumed to stay constant for the entire range of probed tip excursions Δ*z*. Relaxations of the junction geometry are therefore surmised to play a minor role. Indeed, with this assumption, the evolution of ε can satisfactorily be described (Figure 3). Moreover, a change of, e. g., the straight to a tilted tip would cause avoided crossing, which is not observed.

The sign of *J* can be determined as follows. For ϑ = 0° (Figure 3a), the DSF excitation energy decreases with increasing Δ*z* and *J*, which is observed both in the measurements and the simulations. This redshift in the DSF energy is compatible with *J* > 0, in agreement with previous conclusions.^[^ ^47^ ^]^ In the case ϑ > 0° (Figure 3b), the evolution of the DSF energy is likewise in accordance with *J* > 0 and with previous work^[^ ^47^ ^]^ because surmising *J* < 0 leads to an increase of ε starting already from *J* = 0 (Supporting Information, Figure S3), which is at odds with the experimental data where a rather constant DSF energy at elevated *J* ⩾ 0.8 meV is followed by an increase for *J* ⩾ 1.5 meV. For each Δ*z*, the spin excitation energies are matched with the eigenvalues of $\hat{H}$ (Equation (4)) by adjusting *J*. The fits are presented as solid lines in Figure 3. Most importantly, besides the good overall accordance between experimental and simulated data, the crossing and anticrossing behavior for SSF and DSF energy levels is reproduced. Moreover, the resulting *J*(Δ*z*) variation is exponential in a wide range of tunneling distances with a rate that is reasonably comparable with earlier findings reported for normal‐metal Nc–Nc contacts^[^ ^47^ ^]^ (Supporting Information, Figure S5). The eigenvector analysis of $\hat{H}$ (Supporting Information, Figure S3) further reveals that for ϑ = 0° the SSF level splits for *J* > 0 into the weakly decreasing degenerate SSF_1, 2_ and the strongly increasing degenerate SSF_3, 4_ levels. The latter crosses the DSF_1_ energy level at *J* ≈ 2 meV. In the case of ϑ ≠ 0°, the SSF_3_ and DSF_1_ levels mix and, thus, exhibit anticrossing, while the SSF_4_ transition, which at ϑ = 0° was degenerate with SSF_3_, is suppressed. The suppression can be traced to transition matrix elements, which are discussed next.

### 2.4 Transition Matrix Element Effects

The model Hamiltonian, Equation (4), predicts additional spin excitation levels that are apparently absent from the d*I*/d*V* spectra of the experiment. The origin of this observation is assigned to transition matrix elements affecting the measured tunneling current. To see this more clearly, the phenomenological fit parameter *h*_ε_ (Equation (3)) due to Fermi's Golden Rule becomes the squared transition matrix element |*M*_*if*_|^2^, that is, the inelastic contribution to the tunneling current now reads

(10) $$\begin{array}{ccc} I_{i}\left(V\right) & & \propto \sum_{i,f}{\left|M_{if}\right|}^{2}\int \left[f_{t}\left(E-eV-\varepsilon_{if}\right)-f_{s}\left(E\right)\right]\varrho_{t}\\ & & \left(E-eV-\varepsilon_{if}\right)\varrho_{s}\left(E\right)\;dE \end{array}$$

The matrix element *M*_*if*_ links the initial (*i*) and final (*f*) state of the coupled system comprising the inelastic tunneling electron and the molecular spins, where ε_*if*_ denotes the associated spin excitation energy. A simple approach to the transition matrix element is the expression

(11) $$M_{if}=M_{if,t}+M_{if,s}$$

which is the sum of transition matrix elements describing the scattering of the tunneling electron at the Nc tip (*M*_*if*, t_) and at Nc on the surface (*M*_*if*, s_). Before discussing more sophisticated approaches,^[^ ^57^, ^70^ ^]^ it is interesting to see to which extent Equation (11) can describe the observations. To this end, each summand of Equation (11) is expressed as

(12) $$M_{if,k}=t_{k}\langle \Psi_{f}|\hat{s}\cdot {\hat{S}}_{k}\left|\Psi_{i}\right\rangle$$

In agreement with previous studies,^[^ ^49^, ^70^, ^71^ ^]^ the tunneling electron (spin operator $\hat{s}$) is included via an exchange scattering term ($\hat{s}\cdot {\hat{S}}_{k}$), which couples its spin either to the Nc spin at the tip or at the surface. The wave function Ψ_*i*, *f*_ = |φ_*i*, *f*_, ψ_*i*, *f*_〉 denotes the initial and final states of the tunneling electron (φ_*i*, *f*_) and of the molecular spin states (ψ_*i*, *f*_). Therefore, spin transitions can be forbidden by a symmetry mismatch of Ψ_*i*_ and Ψ_*f*_ or by the violation of angular‐momentum conservation. The prefactor *t*_k_ can be compared with the potential scattering parameter that takes into account the Coulomb scattering of the tunneling electron from an Nc molecule without spin flip (Supporting Information). The sign of *t*_k_ can be positive or negative depending on the, respectively, repulsive or attractive scattering potential.^[^ ^70^, ^72^, ^73^, ^74^ ^]^

**Figure 4:**
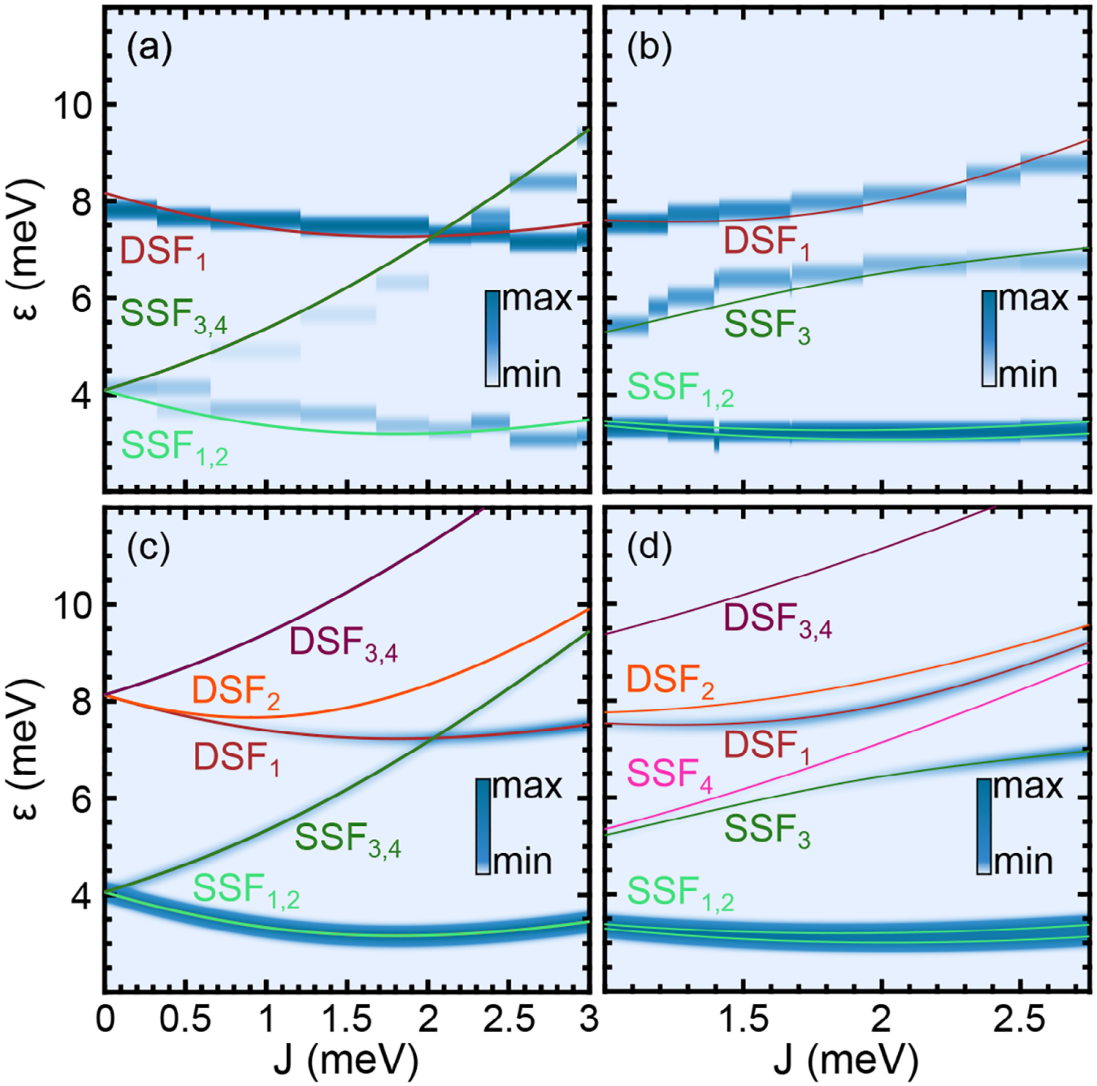
a,b) Strength *h*_ε_ (Equation (3)) of inelastic signals in experimental spin excitation spectra for (a) a straight and (b) a tilted Nc tip encoded by the indicated color scale. c,d) Simulation of |*M*_*if*_|^2^ (Equation (12)) for the indicated spin excitation energy levels for (c) ϑ = 0° with *t*_t_/*t*_s_ = −2 and for (d) ϑ = 21° with *t*_t_/*t*_s_ = −4/3. The color scale is marked.

Experimental signal strengths of the spin excitations in IETS and related squared transition matrix elements are compared in **Figure** **4**. In Figure 4a,b the experimentally obtained *h*_ε_ (Equation (3)) is plotted for the straight (Figure 4a) and a tilted (Figure 4b) Nc tip. The plot of |*M*_*if*_|^2^ (Equation (12)) shows that experimental data can be reproduced in parts. To this end, *t*_t_/*t*_s_ = −2 (*t*_t_/*t*_s_ = −4/3) was identified as the most appropriate ratio for ϑ = 0° (Figure 4c) (ϑ = 21° (Figure 4d)). For instance, in agreement with the experimental data, the DSF_2 − 4_ branches exhibit vanishing |*M*_*if*_|^2^ for straight and tilted Nc tips. In addition, the SSF_4_ level does not appear in the calculations for the tilted Nc tip. There are, however, deviations from the experimental observations, too. At *J* = 0, the simulations only show SSF excitation, while DSF processes are excluded. This is a direct consequence of the model, which exclusively considers single‐exchange excitations, that is, the tunneling electron can flip the spin of either the Nc at the tip or of the adsorbed Nc. Another deviation for ϑ = 0° is the weakness of the DSF_1_ branch compared to the strong SSF_1 − 2_ levels, which contrasts the experimentally observed opposite behavior.

An additional term, ${\hat{S}}_{t}\cdot {\hat{S}}_{s}$, can partially alleviate the presented shortcomings of the model used here (Supporting Information). It allows the flip of both molecular spins while keeping the spin of the tunneling electron invariant.^[^ ^57^ ^]^ The inclusion of ${\hat{S}}_{t}\cdot {\hat{S}}_{s}$ improved the description of experimental data for small *J* (Supporting Information, Figure S6), while the agreement for larger *J* is poor. Indeed, the omission of ${\hat{S}}_{t}\cdot {\hat{S}}_{s}$ in the model used here leads to better results for the elevated‐*J* range. Possibly, the contributions of $\hat{s}\cdot {\hat{S}}_{t}$, $\hat{s}\cdot {\hat{S}}_{s}$, and ${\hat{S}}_{t}\cdot {\hat{S}}_{s}$ depend on the Nc–Nc separation, which future advanced‐level simulations may address.

## 3 Conclusion

Crossing and avoided crossing of quantum spin energy levels occurs in an experimental model system of two exchange‐coupled molecular spins. By perturbing the isotropic interaction between the parallel spin triplets of two adjacent Nc molecules through the tilting of one Nc, the crossing of an SSF and DSF energy level observed from the straight Nc probe turns into an anticrossing for the tilted Nc probe. These findings are relevant to the quantum physical understanding of coupled spin entities. From a nanotechnological perspective they reveal the opportunity of engineering at the ultimate scale a sensitive magnetic probe for exploring nanomagnetism.

## 4 Experimental Section

The experiments were performed with an STM operated in ultrahigh vacuum (5 · 10^−9^ Pa) and at low temperature (5 K). Surfaces of Pb(111) were cleaned and prepared by Ar^+^ ion bombardment and annealing. The clean surface was exposed at 80 K to Nc molecules sublimated from powder (purity: ⩾98.5%) in a ceramics crucible held at room temperature. During Nc deposition the pressure rose to 5 · 10^−7^ Pa. A chemically etched W wire (purity: 99.95 %, diameter: 50 µm) served as the tip material. Field emission on and repeated indentations into the Pb surface led to the coating of the tip apex with substrate material, which was evidenced by the *I*‐*V* characteristics of a superconductor–superconductor tunneling junction. Topographic data were acquired in the constant‐current mode with the voltage applied to the sample and were further processed with WSxM.^[^ ^75^ ^]^ Spectroscopy of d*I*/d*V* proceeded via the sinusoidal modulation (250 µV, 360 Hz) of the dc sample voltage and measuringthe first harmonic of the ac current response of the tunneling junction with a lock‐in amplifier. The transfer of a single Nc molecule from the Pb(111) surface to the tip apex proceeded via deactivating the feedback loop at ±100 mV and 30 pA to 50 pA, changing the sample voltage to −50 mV, and approaching the pristine Pb tip to the center of an adsorbed Nc molecule embedded in a molecular island by 500 pm.

### Statistical Analysis

The presented data were not preprocessed. The given uncertainty margins reflect the 1 % decrease of the reliability factor underlying the least‐squares fits performed with MATLAB.^[^ ^76^ ^]^ The presented microscopic and spectroscopic data are representative. The reliability of spectroscopic data was ensured by reproducing the expected signature of the BCS energy gap.

## Conflict of Interest

The authors declare no conflict of interest.

## Supporting information

Supporting Information

## Data Availability

The data that support the findings of this study are available from the corresponding author upon reasonable request.

## References

[1] P. Gambardella , S. Rusponi , M. Veronese , S. S. Dhesi , C. Grazioli , A. Dallmeyer , I. Cabria , R. Zeller , P. H. Dederichs , K. Kern , C. Carbone , H. Brune , Science 2003, 300, 1130. 12750516 10.1126/science.1082857

[2] C. J. Milios , A. Vinslava , W. Wernsdorfer , S. Moggach , S. Parsons , S. P. Perlepes , G. Christou , E. K. Brechin , J. Am. Chem. Soc. 2007, 129, 2754. 17309264 10.1021/ja068961m

[3] A. Gaita‐Ariño , F. Luis , S. Hill , E. Coronado , Nat. Chem. 2019, 11, 301. 30903036 10.1038/s41557-019-0232-y

[4] J. M. Zadrozny , J. Niklas , O. G. Poluektov , D. E. Freedman , ACS Cent. Sci. 2015, 1, 488. 27163013 10.1021/acscentsci.5b00338 PMC4827467

[5] Y. Chen , Y. Bae , A. J. Heinrich , Adv. Mater. 2023, 35, 2107534. 10.1002/adma.202107534 34994026

[6] L. Bogani , W. Wernsdorfer , Nat. Mater. 2008, 7, 179. 18297126 10.1038/nmat2133

[7] W. Liang , M. P. Shores , M. Bockrath , J. R. Long , H. Park , Nature 2002, 417, 725. 12066180 10.1038/nature00790

[8] R. Wiesendanger , Nat. Rev. Mater. 2016, 1, 16044.

[9] J. Hermenau , J. Iban̆ez‐Azpiroz , C. Hübner , A. Sonntag , B. Baxevanis , K. T. Ton , M. Steinbrecher , A. A. Khajetoorians , M. dos Santos Dias , S. Blügel , R. Wiesendanger , S. Lounis , J. Wiebe , Nat. Commun. 2017, 8, 642. 28935897 10.1038/s41467-017-00506-7 PMC5608713

[10] B. Kiraly , A. N. Rudenko , W. M. J. van Weerdenburg , D. Wegner , M. I. Katsnelson , A. A. Khajetoorians , Nat. Commun. 2018, 9, 3904. 30254221 10.1038/s41467-018-06337-4 PMC6156418

[11] A. J. Heinrich , J. A. Gupta , C. P. Lutz , D. M. Eigler , Science 2004, 306, 466. 15358866 10.1126/science.1101077

[12] C. F. Hirjibehedin , C. P. Lutz , A. J. Heinrich , Science 2006, 312, 1021. 16574821 10.1126/science.1125398

[13] A. Spinelli , B. Bryant , F. Delgado , J. Fernández‐Rossier , A. F. Otte , Nat. Mater. 2014, 13, 782. 24997736 10.1038/nmat4018

[14] S. Baumann , F. Donati , S. Stepanow , S. Rusponi , W. Paul , S. Gangopadhyay , I. G. Rau , G. E. Pacchioni , L. Gragnaniello , M. Pivetta , J. Dreiser , C. Piamonteze , C. P. Lutz , R. M. Macfarlane , B. A. Jones , P. Gambardella , A. J. Heinrich , H. Brune , Phys. Rev. Lett. 2015, 115, 237202. 26684139 10.1103/PhysRevLett.115.237202

[15] S. Baumann , W. Paul , T. Choi , C. P. Lutz , A. Ardavan , A. J. Heinrich , Science 2015, 350, 417. 26494753 10.1126/science.aac8703

[16] H. J. Lee , W. Ho , M. Persson , Phys. Rev. Lett. 2004, 92, 186802. 15169520 10.1103/PhysRevLett.92.186802

[17] D. Kitchen , A. Richardella , J.‐M. Tang , M. E. Flatté , A. Yazdani , Nature 2006, 442, 436. 16871214 10.1038/nature04971

[18] P. Wahl , P. Simon , L. Diekhöner , V. S. Stepanyuk , P. Bruno , M. A. Schneider , K. Kern , Phys. Rev. Lett. 2007, 98, 056601. 17358878 10.1103/PhysRevLett.98.056601

[19] T. Uchihashi , J. Zhang , J. Kröger , R. Berndt , Phys. Rev. B 2008, 78, 033402.

[20] N. Néel , J. Kröger , R. Berndt , Phys. Rev. B 2010, 82, 233401.

[21] N. Néel , R. Berndt , J. Kröger , T. O. Wehling , A. I. Lichtenstein , M. I. Katsnelson , Phys. Rev. Lett. 2011, 107, 106804. 21981521 10.1103/PhysRevLett.107.106804

[22] H. Prüser , P. E. Dargel , M. Bouhassoune , R. G. Ulbrich , T. Pruschke , S. Lounis , M. Wenderoth , Nat. Commun. 2014, 5, 5417. 25384417 10.1038/ncomms6417

[23] A. Spinelli , M. Gerrits , R. Toskovic , B. Bryant , M. Ternes , A. F. Otte , Nat. Commun. 2015, 6, 10046. 26616044 10.1038/ncomms10046 PMC4674668

[24] T. Esat , B. Lechtenberg , T. Deilmann , C. Wagner , P. Krüger , R. Temirov , M. Rohlfing , F. B. Anders , F. S. Tautz , Nat. Phys. 2016, 12, 867.

[25] M. Bode , S. Heinze , A. Kubetzka , O. Pietzsch , X. Nie , G. Bihlmayer , S. Blügel , R. Wiesendanger , Phys. Rev. Lett. 2002, 89, 237205. 12485038 10.1103/PhysRevLett.89.237205

[26] N. Néel , J. Kröger , R. Berndt , Phys. Rev. Lett. 2009, 102, 086805. 19257770 10.1103/PhysRevLett.102.086805

[27] K. Tao , I. Rungger , S. Sanvito , V. S. Stepanyuk , Phys. Rev. B 2010, 82, 085412. 10.1103/PhysRevLett.103.057202 19792529

[28] M. Ziegler , N. Néel , C. Lazo , P. Ferriani , S. Heinze , J. Kröger , R. Berndt , New J. Phys. 2011, 13, 085011.

[29] S. Schmaus , A. Bagrets , Y. Nahas , T. K. Yamada , A. Bork , M. Bowen , E. Beaurepaire , F. Evers , W. Wulfhekel , Nat. Nanotechnol. 2011, 6, 185. 21336269 10.1038/nnano.2011.11

[30] C. Lazo , N. Néel , J. Kröger , R. Berndt , S. Heinze , Phys. Rev. B 2012, 86, 180406.

[31] N. Néel , S. Schröder , N. Ruppelt , P. Ferriani , J. Kröger , R. Berndt , S. Heinze , Phys. Rev. Lett. 2013, 110, 037202. 23373948 10.1103/PhysRevLett.110.037202

[32] J. Schöneberg , F. Otte , N. Néel , A. Weismann , Y. Mokrousov , J. Kröger , R. Berndt , S. Heinze , Nano Lett. 2016, 16, 1450. 26783634 10.1021/acs.nanolett.5b05071

[33] S. Mishra , G. Catarina , F. Wu , R. Ortiz , D. Jacob , K. Eimre , J. Ma , C. A. Pignedoli , X. Feng , P. Ruffieux , J. Fernández‐Rossier , R. Fasel , Nature 2021, 598, 287. 34645998 10.1038/s41586-021-03842-3

[34] J. Hieulle , S. Castro , N. Friedrich , A. Vegliante , F. R. Lara , S. Sanz , D. Rey , M. Corso , T. Frederiksen , J. I. Pascual , D. Peña , Angew. Chem., Int. Ed. 2021, 60, 25224. 10.1002/anie.202108301 PMC9292598 34647398

[35] A. Vegliante , S. Fernández , R. Ortiz , M. Vilas‐Varela , T. Y. Baum , N. Friedrich , F. Romero‐Lara , A. Aguirre , K. Vaxevani , D. Wang , C. Garcia Fernandez , H. S. J. van der Zant , T. Frederiksen , D. Peña , J. I. Pascual , ACS Nano 2024, 18, 26514. 39304184 10.1021/acsnano.4c01963 PMC11448760

[36] T. Choi , W. Paul , S. Rolf‐Pissarczyk , A. J. Macdonald , F. D. Natterer , K. Yang , P. Willke , C. P. Lutz , A. J. Heinrich , Nat. Nanotechnol. 2017, 12, 420. 28263962 10.1038/nnano.2017.18

[37] K. Yang , W. Paul , S.‐H. Phark , P. Willke , Y. Bae , T. Choi , T. Esat , A. Ardavan , A. J. Heinrich , C. P. Lutz , Science 2019, 366, 509. 31649202 10.1126/science.aay6779

[38] X. Zhang , C. Wolf , Y. Wang , H. Aubin , T. Bilgeri , P. Willke , A. J. Heinrich , T. Choi , Nat. Chem. 2022, 14, 59. 34764471 10.1038/s41557-021-00827-7

[39] S.‐H. Ji , T. Zhang , Y.‐S. Fu , X. Chen , X.‐C. Ma , J. Li , W.‐H. Duan , J.‐F. Jia , Q.‐K. Xue , Phys. Rev. Lett. 2008, 100, 226801. 18643441 10.1103/PhysRevLett.100.226801

[40] S. Kezilebieke , M. Dvorak , T. Ojanen , P. Liljeroth , Nano Lett. 2018, 18, 2311. 29533636 10.1021/acs.nanolett.7b05050 PMC6095633

[41] M. Ruby , B. W. Heinrich , Y. Peng , F. von Oppen , K. J. Franke , Phys. Rev. Lett. 2018, 120, 156803. 29756863 10.1103/PhysRevLett.120.156803

[42] D.‐J. Choi , C. G. Fernández , E. Herrera , C. Rubio‐Verdú , M. M. Ugeda , I. Guillamón , H. Suderow , J. I. Pascual , N. Lorente , Phys. Rev. Lett. 2018, 120, 167001. 29756947 10.1103/PhysRevLett.120.167001

[43] L. Zhou , J. Wiebe , S. Lounis , E. Vedmedenko , F. Meier , S. Blügel , P. H. Dederichs , R. Wiesendanger , Nat. Phys. 2010, 6, 187.

[44] A. A. Khajetoorians , J. Wiebe , B. Chilian , S. Lounis , S. Blügel , R. Wiesendanger , Nat. Phys. 2012, 8, 497.

[45] M. Ziegler , N. Ruppelt , N. Néel , J. Kröger , R. Berndt , Appl. Phys. Lett. 2010, 96, 132505.

[46] K. F. Kelly , D. Sarkar , S. Prato , J. S. Resh , G. D. Hale , N. J. Halas , J. Vac. Sci. Technol. B 1996, 14, 593.

[47] G. Czap , P. J. Wagner , F. Xue , L. Gu , J. Li , J. Yao , R. Wu , W. Ho , Science 2019, 364, 670. 31097665 10.1126/science.aaw7505

[48] B. Verlhac , N. Bachellier , L. Garnier , M. Ormaza , P. Abufager , R. Robles , M.‐L. Bocquet , M. Ternes , N. Lorente , L. Limot , Science 2019, 366, 623. 31672895 10.1126/science.aax8222

[49] M. Kögler , N. Néel , L. Limot , J. Kröger , Nano Lett. 2024, 24, 14355. 39475061 10.1021/acs.nanolett.4c04075 PMC11566111

[50] J. Bork , Y.‐h. Zhang , L. Diekhöner , L. Borda , P. Simon , J. Kroha , P. Wahl , K. Kern , Nat. Phys. 2011, 7, 901.

[51] M. Ternes , C. P. Lutz , A. J. Heinrich , W.‐D. Schneider , Phys. Rev. Lett. 2020, 124, 167202. 32383899 10.1103/PhysRevLett.124.167202

[52] M. Ormaza , N. Bachellier , M. N. Faraggi , B. Verlhac , P. Abufager , P. Ohresser , L. Joly , M. Romeo , F. Scheurer , M.‐L. Bocquet , N. Lorente , L. Limot , Nano Lett. 2017, 17, 1877. 28199115 10.1021/acs.nanolett.6b05204

[53] L. Garnier , B. Verlhac , P. Abufager , N. Lorente , M. Ormaza , L. Limot , Nano Lett. 2020, 20, 8193. 33119321 10.1021/acs.nanolett.0c03271

[54] C. Wäckerlin , A. Cahlík , J. Goikoetxea , O. Stetsovych , D. Medvedeva , J. Redondo , M. S̆vec , B. Delley , M. Ondrác̆ek , A. Pinar , M. Blanco‐Rey , J. Kolorenc̆ , A. Arnau , P. Jelínek , ACS Nano 2022, 16, 16402. 36200735 10.1021/acsnano.2c05609

[55] A. Fétida , O. Bengone , M. Romeo , F. Scheurer , R. Robles , N. Lorente , L. Limot , ACS Nano 2024, 18, 13829. 38739416 10.1021/acsnano.4c02470

[56] C. Mier , B. Verlhac , L. Garnier , R. Robles , L. Limot , N. Lorente , D.‐J. Choi , J. Phys. Chem. Lett. 2021, 12, 2983. 33730501 10.1021/acs.jpclett.1c00328

[57] Y. Bae , M. Ternes , K. Yang , A. J. Heinrich , C. Wolf , C. P. Lutz , ACS Nano 2025, 19, 1361. 39810379 10.1021/acsnano.4c13934

[58] M. Ternes , W.‐D. Schneider , J.‐C. Cuevas , C. P. Lutz , C. F. Hirjibehedin , A. J. Heinrich , Phys. Rev. B 2006, 74, 132501.

[59] A. F. Andreev , Sov. Phys. JETP 1964, 19, 1228.

[60] P. de Gennes , D. Saint‐James , Phys. Lett. 1963, 4, 151.

[61] G. Deutscher , Rev. Mod. Phys. 2005, 77, 109.

[62] L. Yu , Acta Phys. Sin. 1965, 21, 75.

[63] H. Shiba , Prog. Theor. Phys. 1968, 40, 435.

[64] A. I. Rusinov , JETP Lett. 1969, 9, 85.

[65] R. C. Dynes , V. Narayanamurti , J. P. Garno , Phys. Rev. Lett. 1978, 41, 1509.

[66] J. Klein , A. Léger , M. Belin , D. Défourneau , M. J. L. Sangster , Phys. Rev. B 1973, 7, 2336.

[67] J. Kröger , L. Limot , H. Jensen , R. Berndt , S. Crampin , E. Pehlke , Prog. Surf. Sci. 2005, 80, 26.

[68] P. Townsend , J. Sutton , Phys. Rev. 1962, 128, 591.

[69] D. Gatteschi , R. Sessoli , J. Villain , Molecular Nanomagnets , Oxford, 2006.

[70] M. Ternes , New J. Phys. 2015, 17, 063016.

[71] P. Jacobson , T. Herden , M. Muenks , G. Laskin , O. Brovko , V. Stepanyuk , M. Ternes , K. Kern , Nat. Commun. 2015, 6, 8536. 26456084 10.1038/ncomms9536 PMC4633813

[72] D. Jacob , Phys. Rev. B 2018, 97, 075428.

[73] C. Rubio‐Verdú , A. Sarasola , D.‐J. Choi , Z. Majzik , R. Ebeling , M. R. Calvo , M. M. Ugeda , A. Garcia‐Lekue , D. Sánchez‐Portal , J. I. Pascual , Commun. Phys. 2018, 1, 15.

[74] J. Li , N. Friedrich , N. Merino , D. G. de Oteyza , D. Peña , D. Jacob , J. I. Pascual , Nano Lett. 2019, 19, 3288. 30964303 10.1021/acs.nanolett.9b00883

[75] I. Horcas , R. Fernández , J. M. Gómez‐Rodríguez , J. Colchero , J. Gómez‐Herrero , A. M. Baro , Rev. Sci. Instrum. 2007, 78, 013705. 17503926 10.1063/1.2432410

[76] T. M. Inc., Matlab version: 23.2 (r2023b), 2023, https://www.mathworks.com.

