# Supplementary material for: Crossing and Anticrossing of Exchange‐Coupled Molecular Spin Excitation Energy Levels
Source: Small. 2025 May 27;22(12):2412703. doi: 10.1002/smll.202412703 (PMC12934386; doi:10.1002/smll.202412703)
Supplement: Supplementary file 1 — Supporting Information [file SMLL-22-2412703-s001.pdf]

Supporting Information: Crossing and anticrossing of exchange-coupled molecular spin excitation energy levels

Lorenz Meyer, Maximilian Kögler, Robert Henninger, Nicolas Néel, Jörg Kröger

Institut für Physik, Technische Universität Ilmenau, D-98693 Ilmenau, Germany

S1 Estimation of the molecular-probe tilt angle

Deviating from previous experiments where geometric details of molecular probes were determined from images of an adsorbed atom [1, 2, 3, 4, 5, 6, 7], the tilt angle of Nc at the STM tip was estimated here by images of adsorbed Nc molecules. Figure S1a shows a cross-sectional profile of an STM image of adsorbed Nc acquired with a pristine Pb tip. The two maxima are associated with the overlap of p_z-orbitals of the Cp group of Nc with Pb orbitals of the tip (Figure S1b), in agreement with observations from Ag(110) [4], Cu(100) [3, 5, 8, 9, 10, 11], and Cu(111) [7]. Their fit with two Gaussians (dashed lines) provides an estimate for the lateral dimension of adsorbed Nc as the separation of the two Gaussian peak positions (≈ 0.5 nm), which is needed for the estimation of the tilt angle of Nc-terminated tips.

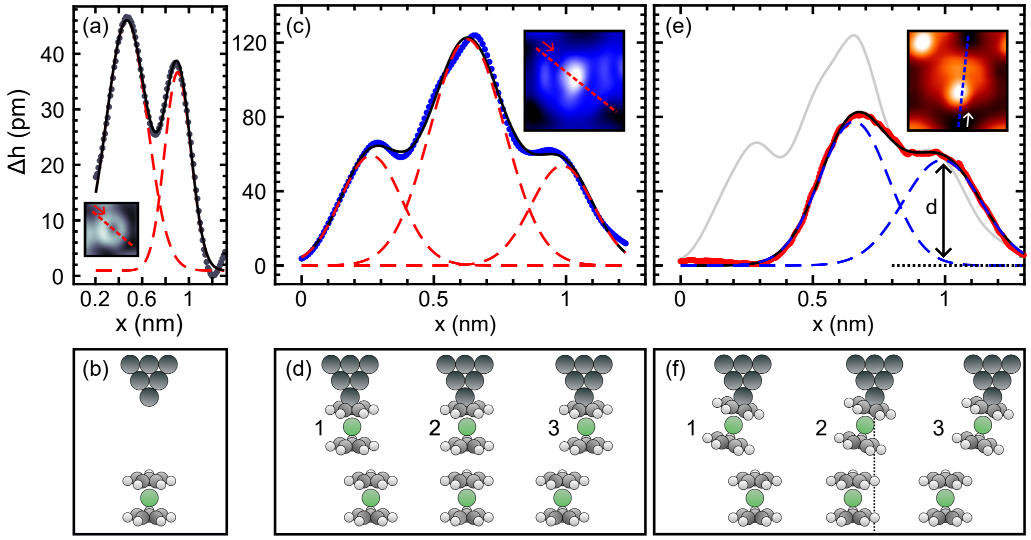

Figure S1: (a) Cross-sectional profile (dots) acquired across the center of an adsorbed Nc with a pristine Pb tip. The solid line depicts the fit of the topographic data with two Gaussians (dashed lines). Inset: STM image of a single Nc acquired with a pristine Pb tip (-100 mV, 30 pA) showing the path (dashed line) of the cross-sectional profile in (a). (b) Sketch of the junction in (a). (c) As (a) for a straight Nc tip. The solid line depicts the fit of the topographic data (dots) with three Gaussians (dashed lines). Inset: STM image of a single Nc acquired with a straight Nc tip (100 mV, 30 pA) showing the path (dashed line) of the cross-sectional profile in (c). (d) Sketches of the junction geometries 1–3 that lead to the topographic maxima in (c). (e) As (c) for a tilted Nc tip. Inset: STM image of a single Nc acquired with a tilted Nc tip (-100 mV, 25 pA) showing the path (dashed line) of the cross-sectional profile in (e). (f) As (d) for a tilted Nc tip.

Indeed, when imaged with a straight Nc tip, adsorbed Nc appears wider (Figure S1c). The maxima associated with the Cp moiety are now separated by ≈ 0.8 nm. The sketch in Figure S1d explains this effect by the lateral extension of the Nc tip. The protruding p_z-orbitals of Nc at the tip overlap with p_z-orbitals of adsorbed Nc before the pristine Pb tip reaches the region of the Cp ring of adsorbed Nc (1 in Figure S1d). When the Ni atom of the two Nc molecules are aligned with the surface normal (2 in Figure S1d) the STM cross-sectional profile gives rise to the central maximum. At position 3, the

pristine Pb tip is already outside the top Cp group of adsorbed Nc, while the p_z–p_z overlap induces the third maximum in the cross-sectional profile.

This effect of apparent widening must also be considered in STM images of adsorbed Nc with a tilted Nc tip (Figure S1e,f). The cross-sectional profile (Figure S1e) shows an asymmetric behavior of the Cp ring apparent height at opposite sites to the central protrusion, which can be understood by the different Cp–Cp distances in the course of imaging the edges of the Cp group of adsorbed Nc (1 and 3 in Figure S1f). The difference d in apparent heights and the corrected lateral separation Δx of the Cp-associated topographic maxima can be used to estimate the tilt angle of the Nc probe according to $\vartheta = \tan^{-1}(d/\Delta x) \approx 8°$.

S2 Spatially resolved spin excitation spectra

Spectroscopy experiments were performed above different sites of the adsorbed Nc islands with the same Nc tip. Figure S2 shows the evolution of spin excitation energies obtained from the center of an upright adsorbed Nc (Figure S2a) and from a site encircled by four Nc molecules (Figure S2b). Aside from small differences in the energies of the SSF and DSF transitions, their overall evolution with the tip excursion is very similar. In addition, the Nc tilt angles obtained from the fits are consistent, i. e., $23° \pm 3°$ (Figure S2a) and $21° \pm 3°$ (Figure S2b) with uncertainty margins reflecting the 1 % decrease of the reliability factor underlying the least-squares fits to the spin excitation spectra.

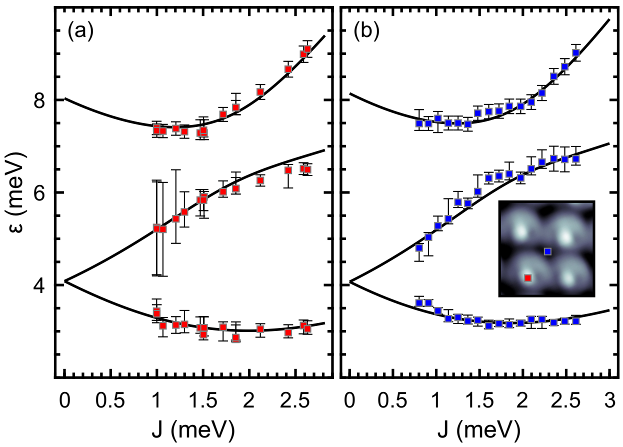

Figure S2: Extracted spin excitation energies ε and their evolution with the intermolecular magnetic exchange coupling J for the same Nc tip positioned (a) above and (b) between Nc molecules in a molecular island. Solid lines in (a) and (b) are fits to the data. Inset to (b): STM image (−100 mV, 25 pA, 1.9 nm × 1.9 nm) of an Nc molecular island with indicated locations for the measurements shown in (a) and (b).

The site of the molecular island giving rise to the data depicted in Figure S2b corresponds to adsorbed Nc molecules that align their Cp planes with the surface normal, i. e., that adopt a lying adsorption configuration [12]. Therefore, the mutual spin orientation of Nc at the tip and lying Nc on the surface is different from the spin alignment in the case of upright adsorbed Nc. A different evolution of the spin excitation energies may hence be expected, which, however, is at odds with the observations, where ε varies in a similar manner for both cases. Possibly, the large diameter of the bottom Cp ring of Nc at the tip probes adjacent upright adsorbed Nc before even reaching the approached lying Nc.

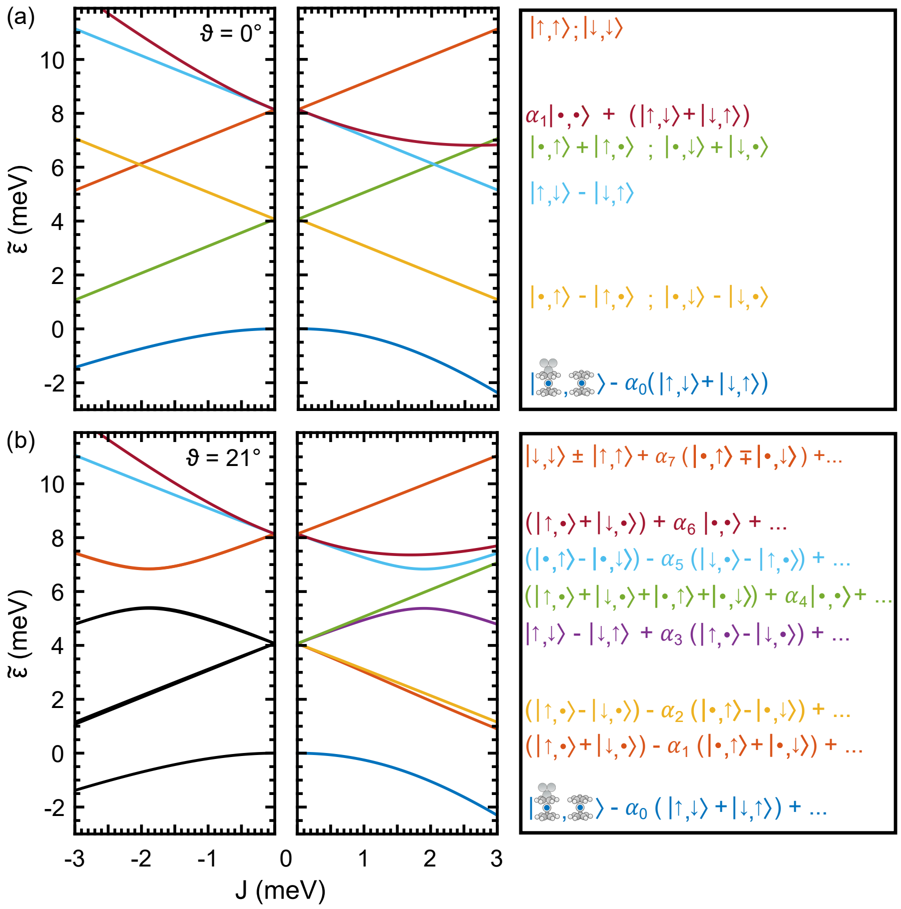

Figure S3: Eigenenergies $\tilde{\varepsilon}$ of $\hat{\mathbf{H}}$ (Equation (4)) for (a) $\vartheta = 0°$, (b) $\vartheta = 21°$ depending on J. The magnetic anisotropies of tip and sample spin were fixed to $D_t(0°) = D_s = 4\,\text{meV}$. The three top branches in (b) depict the evolution of DSF energies with antiparallel (two topmost branches) and parallel (remaining branch) orientation of the Nc spin for low $|J|$. The right panels of (a) and (b) collect eigenvectors $\hat{\mathbf{H}}$ for $J = 3\,\text{meV}$. In both cases, the composition is restricted to the two strongest contributions.

S3 Modulation broadening of the simulated tunneling current

For the description of experimental $\mathrm{d}I/\mathrm{d}V$ spectra, the calculated tunneling current (Equation (1),(3)) is numerically differentiated and convoluted with

$$\chi_m(V) = \begin{cases} \dfrac{2}{\pi}\dfrac{\sqrt{V_m^2 - V^2}}{V_m^2} & \text{for } |V| \leq V_m \\ 0 & \text{for } |V| > V_m \end{cases} \tag{S1}$$

in order to account for the modulation broadening (V_m: amplitude of the ac modulation voltage) [13, 14]. The broadening due to the finite temperature of tip and sample is included in the expression for the current via the Fermi-Dirac function.

S4 Eigenvectors and eigenenergies of the spin Hamiltonian

The main article focuses on the eigenvalues of the spin Hamiltonian $\hat{\mathbf{H}}$ (Equation (4)), which are directly related to the spin excitation energies. In particular, the case $J \geq 0$ is considered. Here, the underlying eigenvectors, i. e., the associated spin states, are presented together with their energy evolution for both signs of J. Figure S3 shows the calculated eigenenergies $\tilde{\varepsilon}$ and eigenvectors of $\hat{\mathbf{H}}$ in the case of a straight ($\vartheta = 0°$, Figure S3a) and a tilted ($\vartheta = 21°$, Figure S3b) Nc tip. The excitation energy ε presented in the

article results from referring $\tilde{\varepsilon}$ to the ground state energy, which is the lowest branch in Figure S3a,b. The eigenvectors (right panels of Figure S3a,b) were evaluated at $J = 3\,\mathrm{meV}$ with the first (second) entry in the ket notation corresponding to the spin state of the Nc tip (adsorbed Nc). The symbols $\bullet$, $\uparrow$, $\downarrow$ refer to $M_S = 0$, 1, -1, respectively. Eigenvectors representing DSF excitations with both Nc spins oriented parallel to the hard axis are excluded in the experiments because of angular-momentum conservation (tunneling electron and Nc spins) [3, 4].

As exposed in the article, owing to the agreement of the experimentally observed and simulated redshift of the DSF excitation energy in the case of $\vartheta = 0°$ (Figure S3a, middle panel), a positive intermolecular exchange coupling J could be inferred. Also for $\vartheta = 21°$ this choice of the sign of J applies, although the experimental data do not conclusively reproduce the DSF energy redshift with increasing $J > 0$ in this case. However, Figure S3b shows that in the case of $J < 0$ the DSF excitations with antiparallel alignment of the molecular spins (two topmost branches) exhibit a blueshift in energy starting already from $J = 0$, which is incompatible with the experimental observations where after a nearly constant DSF energy at elevated J a weak increase follows (Figure 3b).

S5 Perturbative treatment of the level repulsion

In this section, the experimentally observed repulsion of SSF and DSF excitation energy levels is related to the Nc tilt angle in a perturbative approach. For $\vartheta = 0°$ the crossing of $\mathrm{SSF}_{3,4}$ and DSF_1 energy levels occurs at $J = 2\,\mathrm{meV}$ (Figure 3a) with the degenerate energy $\varepsilon^{(0)}$. Diagonalization of $\hat{\mathbf{H}}$ (Equation (4)) gives rise to the underlying eigenvectors

$$|\psi_1\rangle \equiv |\mathrm{SSF}_3\rangle = \frac{1}{\sqrt{2}}\left(|0,-1\rangle + |-1,0\rangle\right) \tag{S2}$$

$$|\psi_2\rangle \equiv |\mathrm{SSF}_4\rangle = \frac{1}{\sqrt{2}}\left(|0,1\rangle + |1,0\rangle\right) \tag{S3}$$

$$|\psi_3\rangle \equiv |\mathrm{DSF}_1\rangle = \frac{1}{\sqrt{2}}\left(|1,-1\rangle - |-1,1\rangle\right) \tag{S4}$$

where the first (second) entry of the ket notation is $M_{S\mathrm{t}}$ ($M_{S\mathrm{s}}$). A finite rotation of the magnetic exchange interaction tensor $\mathbf{J}$ by ϑ introduces a perturbation $\hat{\mathbf{H}}^{(1)}$ to $\hat{\mathbf{H}}(\vartheta = 0°)$, which can readily be calculated using the rotation matrix $\mathbf{R}(\vartheta)$:

$$\hat{\mathbf{H}}^{(1)} = \hat{\mathbf{H}}(\vartheta) - \hat{\mathbf{H}}(\vartheta = 0°) = \hat{\boldsymbol{S}}_\mathrm{t}^\mathrm{T} \cdot \frac{D_\mathrm{t}(0°)}{2} \begin{pmatrix} 0 & 0 & 0 \\ 0 & 2\sin^2\vartheta & -\sin 2\vartheta \\ 0 & -\sin 2\vartheta & -2\sin^2\vartheta \end{pmatrix} \cdot \hat{\boldsymbol{S}}_\mathrm{t} \tag{S5}$$

To examine the influence of $\hat{\mathbf{H}}^{(1)}$ on the spin excitation energy $\varepsilon^{(0)}$, the degenerate subspace spanned by $|\psi_i\rangle$ ($i = 1, 2, 3$) is associated with the block matrix

$$\begin{pmatrix} \cdots & \cdots & \cdots & \cdots & \cdots \\ \cdots & \varepsilon^{(0)} + H_{11}^{(1)} & H_{12}^{(1)*} & H_{13}^{(1)*} & \cdots \\ \cdots & H_{12}^{(1)} & \varepsilon^{(0)} + H_{22}^{(1)} & H_{23}^{(1)*} & \cdots \\ \cdots & H_{13}^{(1)} & H_{23}^{(1)} & \varepsilon^{(0)} + H_{33}^{(1)} & \cdots \\ \cdots & \cdots & \cdots & \cdots & \cdots \end{pmatrix} \approx \begin{pmatrix} \cdots & \cdots & \cdots & \cdots & \cdots \\ \cdots & \varepsilon^{(0)} & 0 & -\mathrm{i}C & \cdots \\ \cdots & 0 & \varepsilon^{(0)} & \mathrm{i}C & \cdots \\ \cdots & \mathrm{i}C & -\mathrm{i}C & \varepsilon^{(0)} & \cdots \\ \cdots & \cdots & \cdots & \cdots & \cdots \end{pmatrix} \tag{S6}$$

of $\hat{\mathbf{H}}$ in the limit of small ϑ, with matrix elements $H_{ij}^{(1)} = \langle\psi_i|\,\hat{\mathbf{H}}^{(1)}\,|\psi_j\rangle$ and the asterisk indicating the complex conjugate. Constant C is expressed as $C = [D_\mathrm{t}(0°)\sin 2\vartheta]/(4\sqrt{2})$. The eigenvalues of this block matrix for $\vartheta \approx 0°$ are $\varepsilon_1^{(1)} = \varepsilon^{(0)}$ and $\varepsilon_\pm^{(1)} = \varepsilon^{(0)} \pm [D_\mathrm{t}(0°)\sin 2\vartheta]/4$. Therefore, the experimentally determined level repulsion

$$\Delta\varepsilon = \varepsilon_+^{(1)} - \varepsilon_-^{(1)} = \frac{D_\mathrm{t}(0°)\sin 2\vartheta}{2} \tag{S7}$$

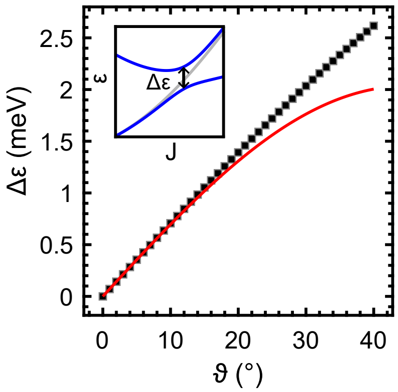

Figure S4: Level repulsion $\Delta\varepsilon$ as a function of the tilt angle ϑ. The graphs of $\Delta\varepsilon(\vartheta)$ obtained from Equation (S7) (solid line) and numerical calculations (squares) are compared. Inset: Illustration of the minimum energy level difference $\Delta\varepsilon$ between the SSF_3 (bottom) and the DSF_1 (top) excitation energies. The spin Hamiltonian underlying the numerical calculations was solved for $D_t(0^\circ) = D_s = 4\,\text{meV}$.

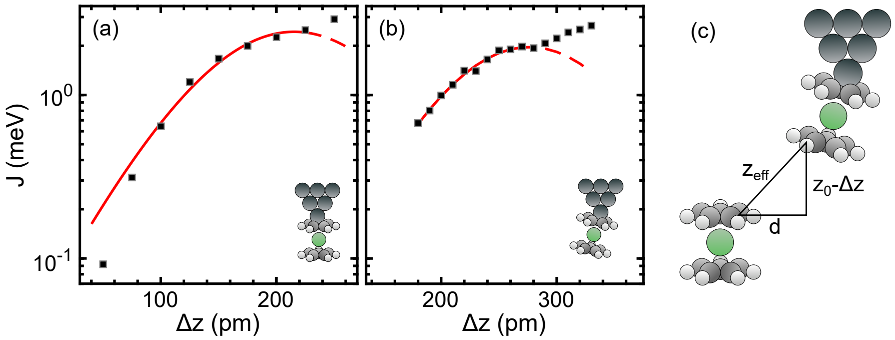

Figure S5: Variation of J with Δz (dots) for (a) a straight and (b) a tilted Nc tip used for acquiring the data presented in, respectively Figure 3a and 3b of the article. The scale for J is logarithmic. The solid and dashed lines represent a fit of Equation (S8) to the data. (c) Sketch of the assumed junction geometry for an off-center Nc tip approach with indicated distances.

fixes the tilt angle of Nc at the tip for the subsequent fits. Figure S4 compares $\Delta\varepsilon(\vartheta)$ according to Equation (S7) (solid line) with the numerical solution (squares). Starting from $\vartheta \approx 20^\circ$ deviations become visible.

The avoided crossing of SSF and DSF excitation energy levels at finite ϑ is due to the intermixing of states, which can be understood in a geometrical picture. For $\vartheta > 0^\circ$, the ground state Nc spin at the tip attains a nonzero z component leading to an admixture of $M_{S_t} = \pm 1$ to the pure SSF level, which in turn adds DSF character.

S6 Distance dependence of the intermolecular exchange coupling

The evolution of the spin excitation energies ε with the tip excursion Δz is described by the spin Hamiltonian $\hat{\mathbf{H}}$ (Equation (4)). To this end, at each Δz the fit parameter J is extracted. Figure S5 shows the results obtained for a straight (Figure S5a) and tilted (Figure S5b) Nc tip approaching a region between four adsorbed Nc molecules (inset to Figure S2b). The magnetic exchange interaction, which is plotted on a logarithmic scale, varies in a more complex manner than the expected uniform exponential behavior. It first rises exponentially and then temporarily levels off in both cases. For the tilted tip, data are available for elevated Δz, which show the further exponential increase, albeit with lower slope than observed from small Δz. In parts, this evolution can be understood by considering the effective distance ($z_{\text{eff}} = \sqrt{d^2 + (z_0 - \Delta z)^2}$, Figure S5c) between the nearest Cp moieties of Nc at the tip

and on the surface, where $d = 200\,\mathrm{pm}$ corresponds to the experimental lateral offset of the Nc tip position. It was previously reported that the π orbitals of the Cp groups mediate the magnetic exchange interaction [4, 5, 15]. The solid and dashed lines in Figure S5a,b depict the graph of

$$J = a \exp\left(-\frac{z_{\mathrm{eff}}}{b}\right) \tag{S8}$$

In the uniform exponential increase of J at low Δz, the extracted parameter b is $26 \pm 8\,\mathrm{pm}$ (Figure S5a) and $20 \pm 7\,\mathrm{pm}$ (Figure S5b), which is comparable to previously reported values ranging from 28.3 pm to 36.5 pm [4]. In addition, Equation (S8) suggests a maximum of J for $\Delta z = z_0$, i. e., when the bottom Cp ring of the Nc tip and the top Cp group of adsorbed Nc are next to each other (Figure S5c). In the experiments, this situation most likely occurs at $\Delta z = 215 \pm 10\,\mathrm{pm}$ (Figure S5a) and $\Delta z = 280 \pm 6\,\mathrm{pm}$ (Figure S5b) where $J(\Delta z)$ temporarily levels off. For $\Delta z > 225\,\mathrm{pm}$ (Figure S5a) and $\Delta z > 310\,\mathrm{pm}$ (Figure S5b) the experimental data clearly deviate from the simulated J (Equation (S8)). Possibly, junction relaxations occur in this range of tip–surface distances, which concomitantly alter orbital overlaps. The appropriate description is reserved to density functional calculations, which are out of the scope of this study.

S7 Transition matrix element effects

The purpose of this section is the application of a recently reported model [15] to the simulation of transition matrix elements together with a comparison to calculations based on the simplified version of this model used in the article.

The inelastic tunneling electron may experience Coulomb scattering at both Nc sites without exchanging angular momentum, or it may induce an Nc spin flip by reversing its own spin. The underlying scattering potential can be expressed as

$$\left(\hat{\boldsymbol{s}} \cdot \hat{\boldsymbol{S}}_{\mathrm{t}} + t_{\mathrm{s}}\right)\left(\hat{\boldsymbol{s}} \cdot \hat{\boldsymbol{S}}_{\mathrm{s}} + t_{\mathrm{t}}\right) + \left(\hat{\boldsymbol{s}} \cdot \hat{\boldsymbol{S}}_{\mathrm{s}} + t_{\mathrm{t}}\right)\left(\hat{\boldsymbol{s}} \cdot \hat{\boldsymbol{S}}_{\mathrm{t}} + t_{\mathrm{s}}\right) = \frac{1}{2}\hat{\boldsymbol{S}}_{\mathrm{s}} \cdot \hat{\boldsymbol{S}}_{\mathrm{t}} + 2\left(t_{\mathrm{t}}\hat{\boldsymbol{s}} \cdot \hat{\boldsymbol{S}}_{\mathrm{t}} + t_{\mathrm{s}}\hat{\boldsymbol{s}} \cdot \hat{\boldsymbol{S}}_{\mathrm{s}} + t_{\mathrm{t}}t_{\mathrm{s}}\right) \tag{S9}$$

where $\hbar$ was set to unity for simplicity. The first term of the right side of Equation (S9) leaves the spin $\hat{\boldsymbol{s}}$ of the tunneling electron invariant and affects both Nc spins, $\hat{\boldsymbol{S}}_{\mathrm{t}}$ and $\hat{\boldsymbol{S}}_{\mathrm{s}}$. The DSF excitation at $J = 0$ is reflected by this expression. The last term marks pure potential scattering with strength t_{t} (t_{s}) at the Nc tip (adsorbed Nc) and gives rise to a constant background conductance across the whole sample voltage range. This term is therefore excluded from the further discussion. The second and third term of Equation (S9) include the exchange scattering between the tunneling electron and the individually addressed Nc spins. Using ladder spin operators ($\hat{s}_{\pm}$, $\hat{S}_{\pm}$), these single-exchange terms can be written as

$$\hat{\boldsymbol{s}} \cdot \hat{\boldsymbol{S}}_{\mathrm{k}} = \hat{s}_z \hat{S}_{\mathrm{k}z} + \frac{1}{2}\left(\hat{s}_+ \hat{S}_{\mathrm{k}-} + \hat{s}_- \hat{S}_{\mathrm{k}+}\right) \tag{S10}$$

with $\mathrm{k} = \mathrm{t}, \mathrm{s}$. These terms reflect for $J = 0$ the spin flip of either the Nc at the tip or the adsorbed Nc with a probability proportional to $|M_{if,\mathrm{k}}|^2 = \left|t_{\mathrm{k}} \langle \Psi_i | \, \hat{\boldsymbol{s}} \cdot \hat{\boldsymbol{S}}_{\mathrm{k}} \, | \Psi_f \rangle\right|^2$. It can be shown that the interference of $M_{if,\mathrm{t}}$ and $M_{if,\mathrm{s}}$ vanishes, while it becomes important for $J \neq 0$.

Recasting Equation (S9) to

$$\hat{\mathbf{h}} \equiv t_0 \hat{\boldsymbol{S}}_{\mathrm{t}} \cdot \hat{\boldsymbol{S}}_{\mathrm{s}} + t_{\mathrm{t}} \hat{\boldsymbol{s}} \cdot \hat{\boldsymbol{S}}_{\mathrm{t}} + t_{\mathrm{s}} \hat{\boldsymbol{s}} \cdot \hat{\boldsymbol{S}}_{\mathrm{s}} \tag{S11}$$

with neglected $t_{\mathrm{t}}t_{\mathrm{s}}$ (vide supra) and constant t_0 controlling the relative contribution of the DSF term $\hat{\boldsymbol{S}}_{\mathrm{t}} \cdot \hat{\boldsymbol{S}}_{\mathrm{s}}$, the signal strength of spin excitations results from the absolute square of the matrix element $M_{if} = \langle \psi_i | \, \hat{\mathbf{h}} \, | \psi_f \rangle$. For $t_0 = 0$, the single-exchange model used in the article is reproduced, which includes the absence of DSF processes for $J = 0$. Figure S6 shows that $t_0 \neq 0$ in Equation (S11) relieves this deficiency. For straight (Figure S6a) and tilted (Figure S6b) Nc tips the DSF excitation is clearly present. However, for $J > 1.5\,\mathrm{meV}$ the DSF_2 excitation is still dominant, which is in contradiction with

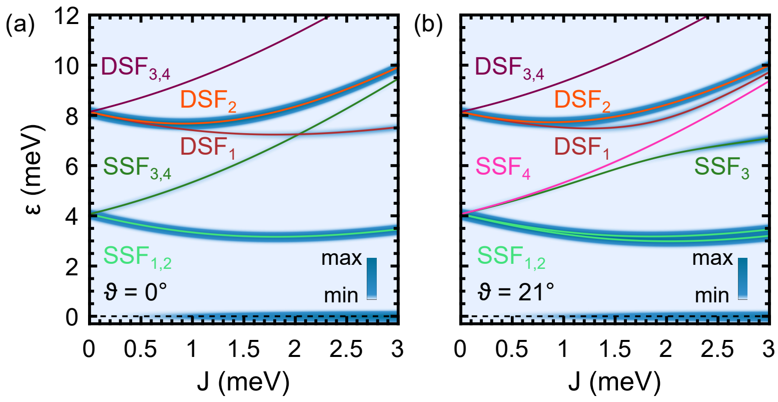

Figure S6: Evaluation of $|M_{if}|^2$ related to $\hat{\mathbf{h}}$ (Equation (S11)) as a function of J for (a) $\vartheta = 0°$, $t_0 = 3t_\mathrm{t}/2 = -3t_\mathrm{s}$ and (b) $\vartheta = 21°$, $t_0 = t_\mathrm{t} = -4t_\mathrm{s}/3$. The color scale is indicated. The simulations are based on $D_\mathrm{t}(0°) = D_\mathrm{s} = 4\,\mathrm{meV}$.

the experimental observation where the DSF_2 level does not even belong to the spectra. Remarkably, the model used in the article, i. e., Equation (S11) with $t_0 = 0$, describes the data for $J > 1.5\,\mathrm{meV}$ very well. Consequently, both approaches appear to be valid for different regions of J. It is therefore tempting to assume that the inelastic processes depend on the tip–surface distance, which controls J and which changes the weights of the three terms in Equation (S11). Moreover, the actual substrate hosting the molecular adsorbate is likely to participate in the spin excitation. Indeed, for Nc–Nc junctions on Ag(110) a weak DSF_2 excitation was reported [4], while on Ag(100) a constantly dominant DSF_2 process was observed [15]. Modeling of distance and substrate dependence of the Nc–Nc spin excitation is reserved to future theory development.

References

[1] K. F. Kelly, D. Sarkar, S. Prato, J. S. Resh, G. D. Hale, N. J. Halas, *J. Vac. Sci. Technol. B* **1996**, *14* 593.

[2] G. Schull, T. Frederiksen, A. Arnau, D. Sanchez-Portal, R. Berndt, *Nat. Nanotechnol.* **2011**, *6* 23.

[3] M. Ormaza, N. Bachellier, M. N. Faraggi, B. Verlhac, P. Abufager, P. Ohresser, L. Joly, M. Romeo, F. Scheurer, M.-L. Bocquet, N. Lorente, L. Limot, *Nano Letters* **2017**, *17* 1877.

[4] G. Czap, P. J. Wagner, F. Xue, L. Gu, J. Li, J. Yao, R. Wu, W. Ho, *Science* **2019**, *364* 670.

[5] B. Verlhac, N. Bachellier, L. Garnier, M. Ormaza, P. Abufager, R. Robles, M.-L. Bocquet, M. Ternes, N. Lorente, L. Limot, *Science* **2019**, *366* 623.

[6] J. Brand, S. Leitherer, N. R. Papior, N. Néel, Y. Lei, M. Brandbyge, J. Kröger, *Nano Letters* **2019**, *19* 7845.

[7] M. Kögler, N. Néel, L. Limot, J. Kröger, *Nano Letters* **2024**, *24* 14355.

[8] N. Bachellier, M. Ormaza, M. Faraggi, B. Verlhac, M. Vérot, T. Le Bahers, M.-L. Bocquet, L. Limot, *Physical Review B* **2016**, *93* 195403.

[9] M. Ormaza, P. Abufager, B. Verlhac, N. Bachellier, M.-L. Bocquet, N. Lorente, L. Limot, *Nature Communications* **2017**, *8* 1974.

[10] C. Wäckerlin, A. Cahlík, J. Goikoetxea, O. Stetsovych, D. Medvedeva, J. Redondo, M. Švec, B. Delley, M. Ondráček, A. Pinar, M. Blanco-Rey, J. Kolorenč, A. Arnau, P. Jelínek, *ACS Nano* **2022**, *16* 16402.

[11] M. Mohr, M. Gruber, A. Weismann, D. Jacob, P. Abufager, N. Lorente, R. Berndt, *Physical Review B* **2020**, *101* 075414.

[12] C. Mier, B. Verlhac, L. Garnier, R. Robles, L. Limot, N. Lorente, D.-J. Choi, *The Journal of Physical Chemistry Letters* **2021**, *12* 2983.

[13] J. Klein, A. Léger, M. Belin, D. Défourneau, M. J. L. Sangster, *Phys. Rev. B* **1973**, *7* 2336.

[14] J. Kröger, L. Limot, H. Jensen, R. Berndt, S. Crampin, E. Pehlke, *Progress in Surface Science* **2005**, *80* 26 .

[15] Y. Bae, M. Ternes, K. Yang, A. J. Heinrich, C. Wolf, C. P. Lutz, *ACS Nano* **2025**, *19* 1361.
